# Modeling the Device Behavior of Biological and Synthetic Nanopores with Reduced Models

**DOI:** 10.3390/e22111259

**Published:** 2020-11-05

**Authors:** Dezső Boda, Mónika Valiskó, Dirk Gillespie

**Affiliations:** 1 Department of Physical Chemistry, University of Pannonia, P.O. Box 158, H-8201 Veszprém, Hungary; valisko@almos.uni-pannon.hu; 2 Department of Physiology and Biophysics, Rush University Medical Center, Chicago, IL 60612, USA; Dirk_Gillespie@rush.edu

**Keywords:** nanopores, ion channels, reduced models, Monte Carlo, classical density functional theory, Poisson-Nernst-Planck

## Abstract

Biological ion channels and synthetic nanopores are responsible for passive transport of ions through a membrane between two compartments. Modeling these ionic currents is especially amenable to reduced models because the device functions of these pores, the relation of input parameters (e.g., applied voltage, bath concentrations) and output parameters (e.g., current, rectification, selectivity), are well defined. Reduced models focus on the physics that produces the device functions (i.e., the physics of how inputs become outputs) rather than the atomic/molecular-scale physics inside the pore. Here, we propose four rules of thumb for constructing good reduced models of ion channels and nanopores. They are about (1) the importance of the axial concentration profiles, (2) the importance of the pore charges, (3) choosing the right explicit degrees of freedom, and (4) creating the proper response functions. We provide examples for how each rule of thumb helps in creating a reduced model of device behavior.

*We dedicate this paper to our distinguished colleague and dear friend, Douglas Henderson (1934–2020).*

## 1. Introduction

When modeling anything, some approximations must be made, usually to make the calculations feasible. For example, molecular dynamics (MD) simulations use Lennard-Jones (LJ) interactions between atoms in lieu of quantum mechanical interactions. This still keeps the all-atomic nature of the simulations, but can now include more than a small number of atoms. Other models coarse-grain the system much more, reducing the physics to simply calculated properties. Electrical circuits are an example; the electrons are never explicitly modeled, nor are the quantum mechanical interactions that produce electrical resistance. Instead, the concept of resistance is reduced to a proportionality factor between the current and voltage, a kind of response function that (phenomenologically) encapsulates complicated physics in a number. A reduced model can thus be very powerful.

In many nanoscale systems, however, it is not a priori clear how (or even if) one can reduce the physics and still get sensible results. In this paper, we would like to better understand and define when and why reduced models work for certain problems, but not for others? (Ion channels can be considered as natural nanopores, a nomenclature often used in the literature. In this work, when we use the term “nanopore”, we mean synthetic ones.) Why do reduced models work well for some biological ion channels and synthetic nanopores What are reduced models and what defines a “good” reduced model? Our attempt to answer these questions is based on the knowledge accumulated over 20 years [1,2,3,4,5,6,7,8,9,10,11,12,13,14,15,16,17,18,19,20,21,22,23,24,25,26,27,28,29,30,31,32,33,34,35,36,37,38,39,40,41,42,43,44,45,46,47,48,49,50,51,52] of modeling and computing permeation and selectivity in ion channels and nanopores.

### 1.1. The Device Approach

Reduced models are especially useful if we focus on a system as a simple device [53,54]. A device is a black box that responds to some incoming stimuli (input signals) by producing output signals. Our goal is to open the black box a little bit and peak into it to understand the inner mechanisms of the device that make the output. In the case of nanodevices, understanding necessarily means understanding molecular mechanisms due to the microscopic dimensions of the underlying processes. This is generally accomplished by modeling. In our model, we do not want to include everything; we focus on those components that are absolutely necessary to reproduce device behavior. By device behavior, we mean the relation of the input signal and output signal, also called device function.

By focusing on device function we reduce the problem at hand. We look at a complex system from an engineering point of view. While complex systems are called complex because the engineering approach tends to fail, there are systems where focusing on the important degrees of freedom allows us to reproduce and explain device behavior, which is an experimentally measurable quantity. The system gives the same response to a given signal in a reproducible manner no matter how complicated the underlying molecular processes are.

Let us take the example of a toy model of an airplane. If we want to reproduce the primary device function—the plane flies as a result of lift produced by a horizontal driving force—we do not need to model unimportant degrees of freedom like seats inside the plane and screens on the backs of the seats. We just need to model the proper shape of the plane, the wings especially. Those are the important degrees of freedom.

Similarly, in modeling ion channels, the knowledge of which amino acid residues are charged (and thus interact with the ions) is an important degree of freedom. The residues that are uncharged and are far from the pore are unimportant. For example, in our model of the 2.2 megadalton ryanodine receptor (RyR) channel (one of the largest ion channels known), we only include five charged amino acids. Moreover, as we describe later in Section 3.1.2, not having the surface charge pattern correct (because all the charged amino acids had not been identified yet) produces qualitatively incorrect results.

### 1.2. Ion Channels and Nanopores as Devices

A basic function of nanopores and open ion channels is to connect the bulk aqueous phases separated by a membrane and let ions through in a controlled manner [55,56,57]. The basic input signals of the baths+pore system are the concentrations and electrical potentials on the two sides of the membrane. A difference in any of these properties (concentration and/or electrical potential difference, for example, voltage) acts as a driving force for the diffusing ions and results in ionic currents that are the output signals of the system.

We can also consider the structural features of the nanopore as an input signal as soon as they can be changed easily. The most important feature is the surface charge pattern on the wall of the nanopore [58]. This can be modified very easily with pH [59,60,61] or an electrical potential [62,63] applied on the wall of a nanopore (a gate potential, to borrow a term from semiconductors) when it is made of a conducting material (typically, gold). Surface charge pattern can also be changed with chemical methods in the case of nanopores [64,65] and with point mutation techniques in the case of ion channels. Here, we restrict ourselves to bath concentrations and voltage (the boundary conditions of the problem of steady state transport) as the main input parameters also controlled by experiments.

The pore’s structural features are important because they determine the current response of the device given to the driving force. That relation determines the various useful device functions that are commonly attributed to ion channels and nanopores. An especially important feature of ion channels is selectivity. Various ion channels with well-defined functions in the cell are often distinguished by the specific ion that they favor over other kinds of ions. Regardless of their strict selectivity properties, ions channels are often named on the basis of their physiological roles in the cell. This way, for example, we distinguish calcium channels, potassium channels, sodium channels, and so on.

Nanopores can be manipulated more easily, so they can exhibit a wider variety of functions [56,65,66,67,68,69,70,71,72]. They can also be selective if they distinguish cations from anions. They can behave as diodes if they let ions through at one sign of the voltage, but not at the opposite sign of voltage, a phenomenon called rectification. If we can modify the pore’s properties by a third signal (gate voltage or pH, for example), we can use the pore as a transistor [45,48,73]. We can also decorate the nanopore’s wall with molecules that bind certain ion species selectively. In this case, if that ion is present in the electrolyte, it can change the pore wall’s properties by binding to these molecules and thus changing the current of the background electrolyte. In this way, the nanopore can be used as a sensor [43,47,49,51]. The range of applications of nanopores for specific tasks is much wider, well beyond the short list above, for example, DNA sequencing.

## 2. Reduced Models

The main idea of reduced models is in their name: the number of degrees of freedom that we treat in detail in the model is reduced. We build only those components into the reduced model that are necessary to reproduce and understand the device function. We call these degrees of freedom the *important* ones. The unimportant or implicit degrees of freedom are treated with less precision and are averaged into “response functions”.

A good reduced model is defined by choosing the important degrees of freedom carefully and constructing sufficiently accurate response functions for the others.

Our aim with this paper is to illustrate how to accomplish this, with ion channels and nanopores as worked examples.

The first question is how reduced our model should be? How much detail can we ignore? In this respect, the models shown in this paper belong to a “no man’s land” between the really detailed all-atom models studied by MD simulations popular in ion channel studies and mean-field continuum models (the Poisson-Nernst-Planck (PNP) theory, for example) popular in nanopore studies. We believe that our position between these two limiting cases is especially suitable to shed light on the nature of good reduced models that are appropriate for a well-specified purpose, namely, studying a device behavior.

First, we explain in a few words, why reduced models can be more suitable for ion channel devices than all-atom models, at least, in certain cases. All-atom, in this context, means that we model all water molecules and every single atom of the protein and the membrane explicitly. There are various problems with these all-atom models. They cannot always cover the physiological parameter range, small voltages or small concentrations, for example. They have sampling issues, specifically regarding the simulation of ionic currents, because this means collecting samples of rare events, for example, ions passing through the pore. The applied force fields might be problematic; they tend to overestimate interactions between multiply charged entities due to missing electronic polarization in the models [74]. Also, the models are based on X-ray structures of the protein that are not always available, and even if they are, the crystal structures often do not represent native functional states. For example, the fact that they have been obtained for a frozen structure calls into question their applicability at room temperature. Such uncertainties might be overcome with reduced models with properly adjusted parameters if the goal is to reproduce the conductance properties of the pore.

Reduced models, as soon as they contain the necessary physics, do not suffer from these shortcomings as much (they have other kinds of shortcomings, naturally). They can be simulated faster, sampled better, and the model contains only the basic physics necessary to reproduce the device behavior. One can spare oneself from computing the unimportant details. What is the important physics and degrees of freedom is always determined by the problem at hand, the intention of the investigator (to what deepness are you interested in the details, for example), and the computational resources. Computation, namely, the simulation method with which we investigate the model is a crucial point of the research, but, from the point of view of the train of thoughts of this discussion, they have secondary importance.

### 2.1. Ionic Distribution in the Pore as a Determining Factor

One aspect of our methodologies, however, is important and should be discussed here. In our work, we usually use the Nernst-Planck (NP) transport equation [75,76] to compute the ionic flux:

(1) $$j_{i}\left(r\right)=-\frac{1}{kT}D_{i}\left(r\right)c_{i}\left(r\right)\nabla \mu_{i}\left(r\right),$$

where $j_{i}\left(r\right)$, $D_{i}\left(r\right)$, $c_{i}\left(r\right)$, and $\mu_{i}\left(r\right)$ are the flux density, the diffusion coefficent profile, the concentration profile, and the electrochemical potential profile of ionic species *i*, respectively. One important principle (rule of thumb) of this paper follows from this equation:
*1. The current carried by an ionic species as a result of a given driving force (conductance) is mainly determined by the axial concentration profile of that species inside the pore.*
One interpretation of this statement is the obvious one that if there are more ions in the pore, they will carry more current. The mechanism can, however, be more subtle than that. Pores working on the basis of excluding certain ions from the pore (sodium channels exclude K${}^{+}$, while nanopores with overlapping double layers exclude the coion) are controlled by depletion zones of these excluded ionic species inside the pore somewhere. These depletion zones of low concentration act as high resistance elements in a equivalent circuit if we imagine the consecutive zones of the pore as resistors connected in series. These ideas will be fleshed out below, in our worked examples.

### 2.2. What Determines Local Concentration Inside the Pore?

The probability that a particle is found at a given position r in the system depends on the potential energy, U(r), and the electrochemical potential, $\mu_{i}\left(r\right)$, of ionic species *i* at r (see the acceptance probability of the particle insertion/deletion step in a Grand Canonical Monte Carlo (GCMC) simulation [29]). The distribution of ions inside the pore, therefore, is influenced by (1) local interactions of the ions with pore charges, other ions, solvent molecules, and confining surfaces, and (2) external parameters such as concentration and electrical potential in the baths (the boundary conditions).

If local interactions dominate (U(r) dominates over $\mu_{i}\left(r\right)$), such as in the crowded selectivity filters of calcium channels (see Section 3.1), the concentration profiles are not so sensitive to boundary conditions. In wide nanopores (Section 3.2 and Section 3.3), on the other hand, changes in voltage or bath concentration can significantly influence the concentration profile. In bipolar nanopores, for example, changing the sign of the voltage reduces the depletion zones of ions even further, reducing current and resulting in a diode behavior.

Of these two factors, however, it is the local interactions that are more important for our discussion. These local effects determine the shape of the concentration profile, where it has peaks and where it has depletion zones. They determine the basic device characteristics of the pore and they determine how the pore responds to changes in the external conditions.

We can narrow what is important more specifically. Because free particles (ions and water) just respond to changes in U(r) and $\mu_{i}\left(r\right)$, it is the features (structure) of the pore that determines device function. Moreover, because the ions are charged, their Coulomb interactions with pore charges are dominant; dipolar and higher-order terms in the multipole expansion are secondary both in strength and range. Concentration profiles, therefore, depend sensitively on the distribution of the pore charges. From all our work on channels and pores [5,6,7,8,9,10,11,12,13,14,15,16,17,18,19,20,21,22,23,24,25,26,27,28,29,30,31,33,34,35,36,37,38,39,40,41,42,43,44,45,46,47,48,49,50,51,52] up to this day, we can conclude the following principle:


*2. We need to build the pore charges into the model properly if we want to reproduce local concentration, and, consequently, device function.*


In summary, pore charges are important degrees of freedom, as is the geometry of the pore (length, radius, shape). But what can we say about important and unimportant degrees of freedom?

### 2.3. Important vs. Unimportant Degrees of Freedom

Charges (monopoles) are the first, and strongest term of the multipole expansion. The second, and weaker term is the dipolar one that appears in the interaction of an ion with water molecules. The big question arises whether we need to take the water molecules into account explicitly (as in all-atom MD models), or can we replace them with response functions such as a dielectric constant or a diffusion coefficient?

The answer to this question also depends on the system at hand. In the case of ion channels, it is obvious that explicit water molecules are crucial in potassium channels; the selectivity of that channel is the result of a subtle balance between the interactions of the permeating ions with the atoms of the selectivity filter and with water molecules [77]. Calcium and sodium channels, however, as our model calculations imply, work on the basis of interactions with charged side chains inside the selectivity filter and volume exclusion (discussed below).

Using implicit water is not even a question in the nanopore world, where they abundantly use transport equations and the PB theory. In this world, there is no argument about the necessity of the implicit water model. Instead, we need to argue about the necessity of sophisticated statistical mechanical methods such as classical density functional theory (DFT) or MC.

Why can water be smeared into an implicit background in one case, but not in the other case? In other words, what decides whether explicit water is an important degree of freedom or not? Or, in general, what decides whether any degree of freedom is important or not? We give an explicit answer to this question that, we hope, will be a general recipe for building reduced models:
*3. Those degrees of freedom are the important ones that depend on the input parameters of the device (voltage and concentration), while those that do not can be replaced by response functions.*
If a component of the system does not change considerably upon, for example, changing the voltage, then this component does not influence the mechanisms by which the model generates an output signal as a response to the input signal.

Let us use implicit water as an example to explain this, as this choice is sometimes controversial. Ions are screened by the surrounding water molecules no matter whether external conditions change or not. Certainly, an applied field or the presence of other ions distort the hydration shell around the ions, so screening is changed by changing voltage or concentration.

The effect of external conditions is small if they are small *relative* to primary effects, for example, to interactions with pore charges. If two degrees of freedom have a large relative difference in how they change with external conditions, then we can make the one with the small response implicit. This is a decision for the modeller, and, eventually, a matter of comparison of the model results with reference data. Reference data are primarily experimental data, but they can also be MD results for all-atom models (results will be shown for both cases).

Implicit water, although the most characteristic, is not the only way of reducing the number of explicit degrees of freedom. We can, for example, model the membrane with a slab between two hard walls. We can model the pore with a cylinder of hard wall. We can model the ion channel only by taking its selectivity filter into account, because that is the region that discriminates between ions. We can model protein side chains in a simplified way by taking only the oxygens of the carboxyl groups into account. There are a plenty of ways to simplify the model, but we need to ask ourselves at every step whether the details we just ignored are important or not.

As in the case of the mean-field PNP theory, it can happen that we ignore too much detail. It is well known that PNP cannot reproduce the selectivity behavior of calcium channels, because ionic correlations and volume exclusion that are so important in the highly charged and crowded selectivity filter of Ca channels are absent in PNP. We cannot use the approximations of PNP even in the case of the relatively wide nanopores if multivalent ions are present. Charge inversion, a feature that is common in charged confined systems with multivalent ions cannot be reproduced with PNP [78].

The bottom line is that we need to balance between too many and too few details when we create a model for a specific purpose. If one is curious about the detailed physics of the coordination of ions at binding sites, the reduced model is too crude. If one is studying a wide nanopore with a 1:1 electrolyte in it, PNP theory is probably all right. There is, however, a wide area in between, where ionic correlations (including finite size) matter, but explicit water does not matter.

### 2.4. What Are Good Response Functions?

If we managed to distinguish between important and less important degrees of freedom, the next step is to decide how to smear the less important ones into response functions. There are various possibilities and it is not always obvious which one we should choose. In this respect, we suggest the following principle.
*4. When we create a response function, we should choose one whose parameters do not depend on external conditions, or, at least, we should minimize that dependence. In other words, those parameters should be transferable as much as possible.* This rule might sound obvious because it seems quite ridiculous to refit the parameters for every state point (different values of input device parameters). A model is a model together with its parameters. If those parameters are not stable, meaning transferable between various state points, the model is probably missing some basic physics.

That is exactly the deeper meaning of the above rule. If the physics of the model is right, then it should describe the properties of the nanopore’s wall or the ion channel’s selectivity filter in a robust way. The model should be the same at another voltage or concentration. If the parameters depend on external conditions, they should do that in a physically well-based and explainable way. Otherwise, it is just an unsystematic fitting on the basis of a useless model. The model is useless in this case because it is unusable for prediction. Transferable parameters are the basis of predictions.

In the following, we present our results for three different case studies that illustrate the rules introduced above.

## 3. Case Studies

In the case studies presented in the following sections the system consists of two baths separated by a membrane that contains a pore connecting the two baths. Two electrodes in the two baths produce electrical potential difference (voltage) that is a part of the driving force of the transport of ions. Also, ionic concentrations can be different on the two sides of the membrane. Concentration difference and voltage add up to create an electrochemical potential difference that is the full driving force in the NP equation (Equation (1)).

In the model of this system we include the two baths, the membrane and the pore. The simulation cell is finite surrounded by a boundary at which different boundary conditions are prescribed for the ionic concentrations and the electrical potential on the two sides of the membrane. The electrolyte is modeled in the implicit water framework with the “Primitive Model” that, given the success of our models, is not so primitive after all.

The ions are modeled as charged hard spheres immersed in a dielectric continuum represented by the dielectric constant ϵ, one of the response functions. The interaction potential is

(2) $$u_{ij}\left(r\right)=\left\{\begin{array}{cc} \infty & \mathrm{if}\;r<R_{i}+R_{j}\\ \frac{1}{4\pi \epsilon_{0}\epsilon }\frac{z_{i}z_{j}e^{2}}{r} & \mathrm{if}\;r\geq R_{i}+R_{j}, \end{array}\right.$$

where $R_{i}$ and $R_{j}$ are the radii of ionic species *i* and *j*, respectively, $z_{i}$ and $z_{j}$ are the valences of ionic species *i* and *j*, respectively, $\epsilon_{0}$ is the permittivity of vacuum, *e* is the elementary charge, and *r* is the distance between the two ions. The solvent also exerts its effect on the ions by hindering their diffusion via friction. This is taken into account by another response function, the diffusion coefficient $D_{i}\left(r\right)$ (see Equation (1)), which may include effects beyond interactions with waters, such as interactions with other ions and the confining geometry.

The membrane and the pore are defined by hard walls for simplicity. The most important difference between the test cases is that the pore is modeled differently in different cases. Basically, the shape of the pore and the representation of pore charges are different. By shape of the pore we mean an R(z) function that defines the hard wall obtained by rotating this function around the *z* axis. The models of pore charges will be described in the different cases.

The models are studied with a hybrid simulation method in which the NP equation is coupled to the Local Equilibrium Monte Carlo (LEMC) method (NP+LEMC). The LEMC method is basically a generalization of the GCMC method [79,80] for the case of non-equilibrium systems where the chemical potential is not necessarily constant, so the system is not in global equilibrium. Instead, the input of the LEMC method is the $\mu_{i}\left(r\right)$ profile, while the output is the $c_{i}\left(r\right)$ profile. In practice, the system is divided into small subvolumes, $V^{\alpha }$, in which the $\mu_{i}^{\alpha }$ is constant (local equilibrium is assumed). The result of the simulation is the concentration in each subvolume, $c_{i}^{\alpha }$. The resulting $\mu_{i}^{\alpha }$ and $c_{i}^{\alpha }$ profiles are substituted into the NP equation providing a flux, $j_{i}^{\alpha }$. An iteration process results in a self consistent $\mu_{i}^{\alpha }$ and $c_{i}^{\alpha }$ pair that produces a flux density satisfying the continuity equation, $\nabla \cdot j_{i}\left(r\right)\;=\;0$. It is an expression for local conservation of mass, while in our calculations we use the integrated form that states that the sum of inward and outward currents in and out of a volume element is zero. Details are found in previous papers [29,37,39,41].

The results of other models and computation methods will also be presented. Specifically, we will show results of DFT coupled to the NP equation and MD simulations for explicit water models. These models and methods will be described at the specific system, where they are used.

### 3.1. The Ryanodine Receptor Calcium Channel

The RyR is a biological ion channel that, in muscle, releases Ca${}^{2+}$ ions from the sarcoplasmic reticulum in response to an influx of Ca${}^{2+}$ through L-type calcium channels. In both cardiac and skeletal muscle cells, that RyR-mediated Ca${}^{2+}$ initiates muscle contraction. While its physiological importance is obvious, RyR is also interesting from a single-channel biophysics point of view. Experimentally, its large current allows for relatively easy single-channel current/voltage (IV) recordings. Theoretically, it is a Ca${}^{2+}$-selective channel, but whose preference for Ca${}^{2+}$ is much lower than the L-type calcium channel, even though they share the same selectivity filter in amino acids.

What makes an ion channel a calcium channel is the abundance of negative carboxyl groups (COO${}^{-}$) in and around the selectivity filter. Generally, four glutamate (E) and/or aspartate (D) amino acids line the selectivity filter, which is a short and narrow region of the pore. An important turning point in the understanding of the physics of Ca${}^{2+}$ versus monovalent cations selectivity was a reduced model by Nonner et al. [4] They imagined the selectivity filter of a calcium channel as a high-density fluid where the two oxygens of each of the four COO${}^{-}$ groups were modeled as independent hard-sphere O${}^{1/2-}$ ions (with radius 0.14 nm). When both Na${}^{+}$ and Ca${}^{2+}$ ions compete for space in this “electric stew” [81], the competition is won by Ca${}^{2+}$ ions because they provide twice the charge of Na${}^{+}$ ions while occupying the same volume (as they have similar Pauling radii).

**Figure 1:**
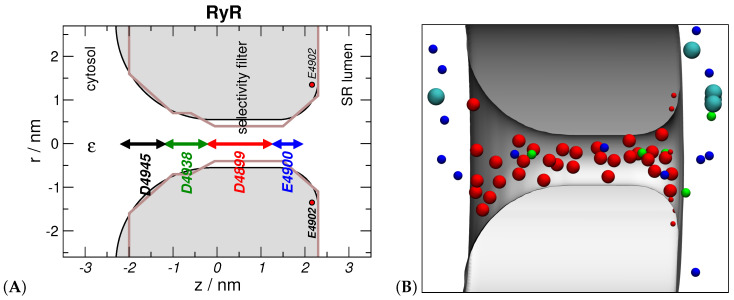
Model of the RyR channel [37]. (**A**) The 3D model is obtained by rotating the shaded gray area about the *z*-axis (the models have rotational symmetry). The arrows indicate the regions into which the 8 O${}^{1/2-}$ ions representing the respective amino acids are confined. The charges of the E4902 residues of the RyR channel are modeled by eight point charges on a ring. The dielectric constants is $\epsilon_{w}\;=\;78.5$ in the whole system. The entire simulation cell is enclosed in a large cylinder. The geometry for the NP+LEMC calculations can be found in Figure 1 of Reference [39]. The brown line indicates the countour of the 1D model of Gillespie [16]. (**B**) A snapshot of the simulation. The blue, green, light blue, and red spheres represent Na${}^{+}$, Ca${}^{2+}$, Cl${}^{-}$, and O${}^{1/2-}$ ions, respectively. This figure was prepared with vmd [82].

This mechanism was later called “Charge-Space Competition” [5] because, while the four negative charges of the selectivity filter attract cations, the crowding of those COO${}^{-}$ groups and the permeating ions inside the very small selectivity filter imposes entropic and energetic penalties for permeating ions (Figure 1). In this scheme, there is a competition between entropic and enthalpic components, creating an advantage for small and/or high-valence cations over large and/or low-valence cations. This effect is amplified when the dielectric constant of the protein surrounding the pore is lower than the dielectric constant of the selectivity filter lumen [13].

When this model of the L-type selectivity filter was incorporated into a pore and studied with GCMC simulations, the model was successful in reproducing the micromolar Ca${}^{2+}$ selectivity of the L-type calcium channel (EEEE locus). Specifically, it reproduced the seminal experiment of Almers and McCleskey [83] where, in 32 mM NaCl, 1 μM Ca${}^{2+}$ in the bath blocks Na${}^{+}$ current, reducing it to half that in the absence of Ca${}^{2+}$. The block works because Ca${}^{2+}$ ions displace Na${}^{+}$ ions in the selectivity filter even though they are present in the bath at much smaller concentrations than the Na${}^{+}$ ions. The model also reproduced [17,21,24] other mole fraction experiments (e.g., Ca${}^{2+}$ vs. Ba${}^{2+}$ [84,85,86], Li${}^{+}$ vs. Na${}^{+}$ [87]) and Gd${}^{3+}$-block of ionic current [88]. Lastly, we were able to interpret [14] the experiments of Heinemann et al. [89] where a DEKA→DEEA mutation converted a sodium channel without a Ca${}^{2+}$ blockade into a calcium channel with $10^{-4}$ M affinity.

Concurrent to this work on the physics of L-type calcium channel selectivity, one of us (DG) created a 1D reduced model of RyR using DFT based on the Nonner et al. independent-O${}^{1/2-}$ model of the COO${}^{-}$ groups [11]. Here, we focus on a second, improved version of this 1D DFT model [16], as it included more charged amino acids that are outside of the selectivity filter yet play an important role in cation permeation [90] (following the second principle of reduced models). The D4945, D4938, D4899, and E4900 amino acids (four copies of each of them due to the homotetrameric RyR structure) were modeled by confining eight half charged oxygen ions, O${}^{1/2-}$ (with radius 0.14 nm), in the regions indicated by arrows in Figure 1. The E4902 amino acids were placed in a ring at the luminal entrance of the pore.

The purpose of this RyR model was to determine whether a reduced model of this channel could reproduce and predict experimental data. (RyR is more useful for this than L-type calcium channel because of the vast amounts of IV data available for RyR.) Both the model [16] and its subsequent applications [19,20,32,35] showed that this is indeed the case, reproducing all the available IV data from the labs of Gerhard Meissner (University of North Carolina, Chapel Hill) and Michael Fill (Rush University Medical Center, Chicago). Moreover, in these papers the model predicted (before confirming experiments were done) a number of counterintuitive and nonlinear selectivity phenomena in RyR.

Later, a 3D reduced model of RyR was created by Boda et al. [37,41]. The purpose of this model was partly to understand the success of the 1D model, trying to define the effects of radial ion distributions that are ignored in the 1D model (which assumed homogeneity in the radial direction). The profile of the pore radius is indicated by the gray shaded area in Figure 1. Here, we focus on the 3D model because it has been less well analyzed in detail and because it uses the same NP+LEMC simulation technique that is also used for the nanopores, described later, that serve as different case studies of reduced models.

Both the 1D and 3D models reproduce dozens of IV curves, some shown in the Supplementary Information for the 3D model. This indicates that both models seem to capture the basics of the RyR device physics in the axial direction. Therefore, we will discuss how each of the principles of reduced models for nanopores works in these RyR models.

#### 3.1.1. Ionic Concentrations and Current

**Figure 2:**
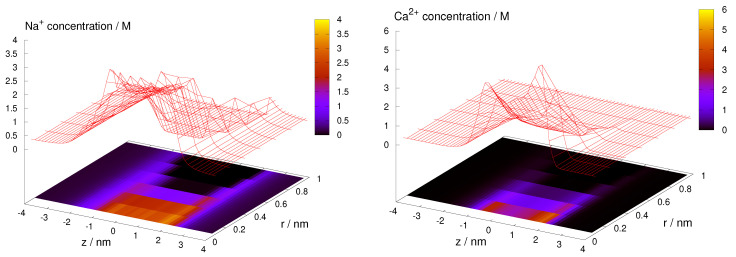
Concentration profiles, $c_{i}\left(z,r\right)$, of Na${}^{+}$ and Ca${}^{2+}$ over the (z,r) plane for 100 mM NaCl and 1 mM CaCl${}_{2}$.

How the ionic profiles determine the species current has several interesting subtleties in RyR. First, given that the 3D model performs equally well as the 1D model, it seems that any radial ion packing effects do not contribute significantly to the current. Figure 2 shows examples for Na${}^{+}$ and Ca${}^{2+}$. The profiles are monotonic in the radial dimension, so the cross-section averaged axial concentration profiles are the main determinants of current. This explains the success of the 1D model.

**Figure 3:**
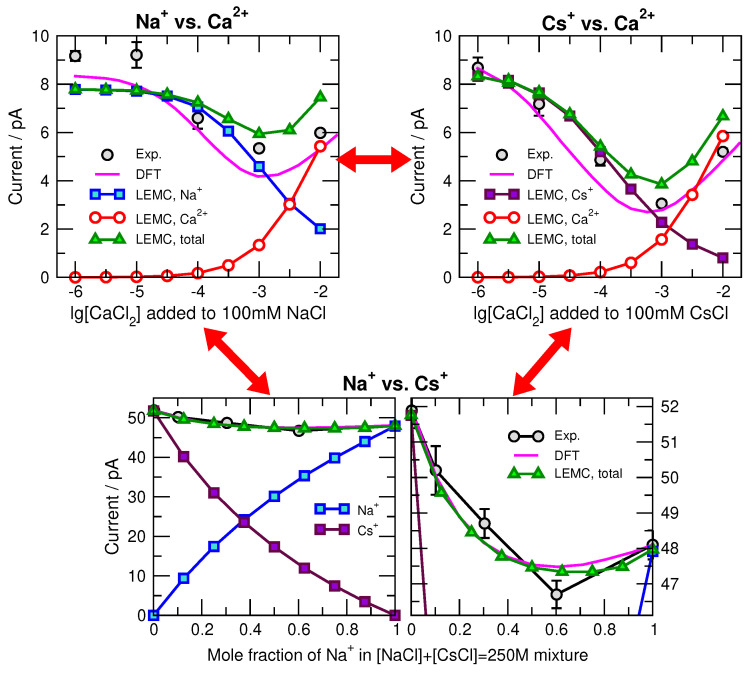
Added-salt experiments for Ca${}^{2+}$ vs. Na${}^{+}$ and Ca${}^{2+}$ vs. Cs${}^{+}$ competition (**top**), and mole-fraction experiments for Na${}^{+}$ vs. Cs${}^{+}$ competition (**bottom**). Details are in the main text.

**Figure 4:**
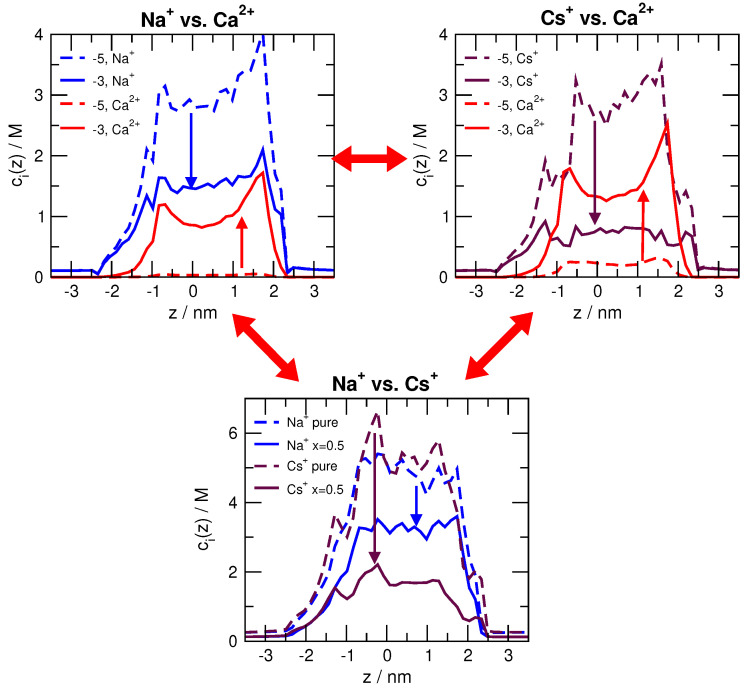
Axial concentration profiles for Ca${}^{2+}$ vs. Na${}^{+}$, Ca${}^{2+}$ vs. Cs${}^{+}$, and Na${}^{+}$ vs. Cs${}^{+}$ competition. In the top row, Ca${}^{2+}$ is added to either Na${}^{+}$ or Cs${}^{+}$. Profiles are for $10^{-5}$ M and $10^{-3}$ M added Ca${}^{2+}$. In the bottom row, the Na${}^{+}$/Cs${}^{+}$ mole fraction profiles are shown for 0, 0.5, and 1 Na${}^{+}$ mole fractions.

Second, high concentration of a species inside the pore does not always translate into high current for that species. This is exemplified in mole fraction experiments, where two cation species compete for the pore (Figure 3 and Figure 4). We distinguish two basic kinds of mole fraction experiments: (1) In one kind, we add one type of cation (e.g., divalents) to a fixed background of the other type of cation (e.g., monovalents), for example, adding CaCl${}_{2}$ to a fixed 100 mM NaCl (or CsCl) solution; (2) In the other kind, we keep the total salt concentration (or ionic strength) fixed while changing the mole fraction of the two salt, for example, a NaCl/CsCl mixture at 250 mM total concentration.

Total current, *I*, or chord conductance, G=I/U (*U* is the applied voltage), can be considered a primary device function in the case of ion channels. But, currents carried by the ionic species are also interesting, and we show those as well. Figure 3 shows the currents as functions of composition expressed either as $\mathrm{lg}[{\mathrm{CaCl}}_{2}]$ for the added-salt experiment or the mole fraction of Na${}^{+}$ ([NaCl] + [CsCl]=250 mM) for the mole fraction experiment.

In the added-Ca${}^{2+}$ experiment with Na${}^{+}$, it is seen that $10^{-3}$ M Ca${}^{2+}$ affects the current; against Na${}^{+}$, RyR has millimolar Ca${}^{2+}$ selectivity. This [Ca${}^{2+}$] is smaller for Cs${}^{+}$ because Ca${}^{2+}$ can compete more easily with the larger Cs${}^{+}$. In both cases, the total current has a minimum, called the anomalous mole fraction effect (AMFE), for experiments (gray spheres), the 1D DFT RyR model (magenta lines), and the 3D NP+LEMC model (green triangles). There is also an AMFE for mixtures of Na${}^{+}$ and Cs${}^{+}$.

To understand the origin of the minimum in current, we first note that at the extremes all the single-species currents are very similar: the all-Na${}^{+}$ current (and all Cs${}^{+}$ current) at $10^{-6}$ M Ca${}^{2+}$ or 0 Cs${}^{+}$ (0 Na${}^{+}$) mole fraction is similar to the all-Ca${}^{2+}$ current at $10^{-2}$ M Ca${}^{2+}$. Why then does current decrease with added Ca${}^{2+}$ (added-salt experiment) or added Na${}^{+}$ (mole fraction experiment) and then increase?

Part of the answer lies in the axial concentration profiles for select Ca${}^{2+}$ concentrations and Na${}^{+}$ mole fractions shown in Figure 4. In the added-salt experiment (top row of figure panels), Ca${}^{2+}$ displaces Na${}^{+}$ and Cs${}^{+}$ throughout the pore. Ca${}^{2+}$ has a much stronger effect on Cs${}^{+}$, indicating that RyR has a higher preference for Ca${}^{2+}$ than Cs${}^{+}$ (compared to Ca${}^{2+}$ versus Na${}^{+}$). Specifically, $10^{-3}$ M Ca${}^{2+}$ displaces almost half the Na${}^{+}$ in the pore and two-thirds of the Cs${}^{+}$. Interestingly, however, the Ca${}^{2+}$ current in Figure 3 (top row) is below the Na${}^{+}$ current and equal to the Cs${}^{+}$ current at $[{\mathrm{Ca}}^{2+}]\;=\;1$ mM. Recall that all single-species currents are nearly identical. Therefore, just because Ca${}^{2+}$ has a large (or even the largest) concentration in the pore, it does not produce as much current as would be predicted from those intra-pore concentrations.

In previous work [17,20], we traced this anomaly to the fact that Ca${}^{2+}$ is at low concentration in the baths, even though it is extremely high (relatively) in the selectivity filter. This produces the counterintuitive result that the bath has a high resistance to Ca${}^{2+}$ flowing, while the selectivity filter has a low resistance. Usually it is the opposite. Only when the bath Ca${}^{2+}$ concentration is relatively high is there an appreciable amount of Ca${}^{2+}$ current. This is physiologically relevant, as resting luminal SR Ca${}^{2+}$ concentration is between 0.5 to 1 mM, and during contractions this is Ca${}^{2+}$ depleted to ∼50 % levels in cardiac myocytes and even lower in skeletal myocytes. The physiological cardiac ion species currents are described in Reference [32].

This is an extreme example of the depletion zones we will discuss for the nanopores later. A depletion zone (a place where ions are absent for the most part) can have as large an effect on current as the regions of high concentration. This is because the axial direction for current flow is made of several regions, the bath, the access region (at the mouth of the channel or pore), the pore, another access region, and another bath. Each of these has a resistance to current flow and the highest resistance element can dominate. In a channel this is usually the selectivity filter because it is commonly physically narrow. However, if it is highly charged, then it will always have ions in it at high concentration and so the bath resistance may dominate the current. In general, the absence of ions in a region can be as consequential as high concentrations.

#### 3.1.2. Accurate Representation of Pore Charges is Important for Reproducing Device Function

As stated above, the first 1D RyR model [11] did not include all the charged groups that the second one [16] does. In fact, it originally included only the two then-known charged groups (Asp-4899 and Glu-4900). But, no parameters could be found to make the computed IV curves resemble, even qualitatively, the experimental curves. Only by hypothesizing the existence of a region of negative charge on the cytosolic side of the selectivity filter did the curves begin to match up. Later, it was determined that two other aspartate groups (Asp-4945 and Asp-4938) also significantly affect ion permeation and selectivity [90]. Only with the explicit addition of these and another charged group (Glu-4902) did the model reproduce all the experimental data and predict even more (which were later confirmed by experiments [16,19,20,32,35]).

#### 3.1.3. Important versus Unimportant Degrees of Freedom

The results of both the 1D and 3D models indicate that the essential important degrees of freedom were captured. One that was left out was ion dehydration. This is crucial for the physiological function of potassium channels [77,91] and excludes Mg${}^{2+}$ from many other calcium channels [92]. However, in RyR it does not seem to play a role, as indicated by both experiments and the models. In experiments, Mg${}^{2+}$ (which has a very large ion dehydration energy compared to the otherwise similar Ca${}^{2+}$) permeates RyR equally as well as Ca${}^{2+}$, indicating no large energetic barrier for Mg${}^{2+}$ entry by stripping off waters. In the two models, missing an important piece of physics ought to result in (large) deviations from the experimental data, especially in Mg${}^{2+}$ versus monovalent cation competition experiments. That this was not seen implies (but does not prove) that ion dehydration is not significant for RyR.

One degree of freedom we have in both the 1D and 3D models that may be superfluous is the flexibility of the O${}^{1/2-}$ to move within their regions of confinement. Our previous work on the L-type calcium channel [27] indicates that their movement in response to other ions being nearby is unimportant for selectivity. Specifically, for that model pore the selectivity behavior of the channel does not change much if we fix the positions of the O${}^{1/2-}$ ions. Seemingly, the important characteristics is the density of the O${}^{1/2-}$ ions inside the pore, while their exact position is secondary. We continue to include the flexibility because it is easy to include and extensive studies would be needed to verify that it is indeed superfluous.

#### 3.1.4. Transferability of Parameters

The main parameters we had that were not based on known RyR structure and that had to be fitted to data were the ionic diffusion coefficients. For both the 1D and 3D models, after these were fit, they were never changed. Therefore, they were used at low and high ionic bath concentrations, low/high and negative/positive applied voltages, and in ionic mixtures. This indicates that they truly are transferable and independent of external conditions.

The one caveat to that statement relates to one of the differences in constructing the 1D and 3D models. In the 3D model, we used only one adjustable $D_{i}^{\mathrm{pore}}$ value in the selectivity filter and interpolated in the vestibules to the bulk. (Values are shown in Table 1.) In the 1D model, on the other hand, there were fitted diffusion coefficients not only in the selectivity filter, but also in the vestibules on either side, in the D4938 and E4900 regions (Figure 1). These were fit for K${}^{+}$ based on data of RyR in symmetric 0.25 M KCl for native RyR (i.e., fully charged) and two charge-neutralizing mutations (D4938N and E4900Q). With these, the 1D model reproduces the nonlinear IV curve of another charge-neutralizing mutation (D4899N) that was not used in fitting the diffusion coefficients. This further shows the transferability of the diffusion coefficients. (All non-K${}^{+}$ cation species were fitted with one experimental data point for the selectivity filter diffusion coefficient and the vestibule values were determined from ratios of the K${}^{+}$ diffusion coefficients in different areas of the pore.)

The 3D model, on the other hand, does not reproduce these charge-neutralizing experiments (data not shown). Therefore, its diffusion coefficients are not as robust against changes to external conditions (although such mutations are large perturbations). This indicates that caution is always in order when interpreting a reduced model outside its established (i.e., tested against experiments) range of external conditions.

### 3.2. Nanopores of Different Device Functions from Different Charge Patterns

**Figure 5:**
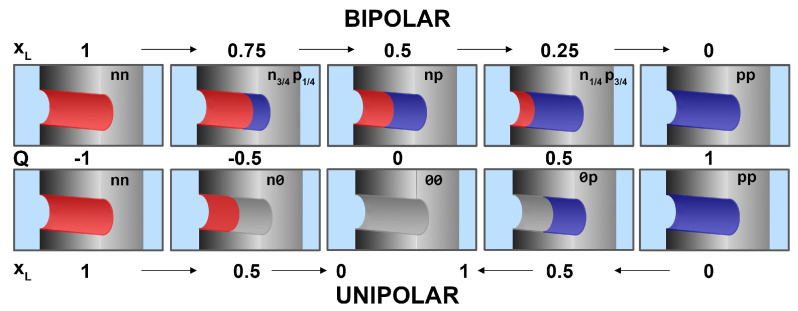
Schematics of the cylindrical nanopores with different charge patterns. There are two regions of lengths $H_{L}$ and $H_{R}$ carrying $\sigma_{L}$ and $\sigma_{R}$ surface charges. We consider either bipolar (top row) or unipolar (bottom row) nanopores. In the bipolar cases, the left-hand region is always negative ($\sigma_{L}\;=\;-\sigma_{0}$ with $\sigma_{0}\;=\;0.4835$*e*/nm${}^{2}$), while the right-hand region is positive ($\sigma_{R}\;=\;\sigma_{0}$). In the unipolar cases, the same is true, but the other side is neutral. The dimensionless net charge, *Q*, increases from left to right, while the fraction of the left region, $x_{L}$ (Equation (3)), changes as indicated by the arrows.

In a recent work [46], we considered synthetic nanopores with varying charge patterns on their walls along the *z*-axis (Figure 5). Although our rules of thumb were not formulated explicitly back then, we practically organized that study along the lines of the four rules of thumb:

- We studied how different charge patterns influence concentration profiles, and, through those, device functions (rules of thumb #1 and #2).
- We performed simulations with models of different resolutions and studied the performance of reduced models compared to all-atom MD simulations. Special attention was given to whether water molecules could be treated implicitly, that is, whether they proved to be “unimportant” degrees of freedom (rule of thumb #3).
- We fit the diffusion coefficients in the pore to MD data and investigated their transferability over varying charge patterns (rule of thumb #4).

A cylindrical nanopore was considered with radius $R_{\mathrm{pore}}\;=\;0.97$ nm and length H=6.4 nm. The wall of the pore was divided into two regions along the *z*-axis: a left (L) region of length $H_{L}$ carrying $\sigma_{L}$ surface charge, and a right (R) region of length $H_{R}\;=\;H-H_{L}$ carrying $\sigma_{R}$ surface charge. The geometry can be characterized by the dimensionless parameter $x_{L}\;=\;H_{L}/H$. We gradually increased $H_{L}$, while keeping the total length, *H*, fixed, so we increased $x_{L}$ from 0 to 1. We performed two series of calculations.

 **Bipolar pores:** The $H_{L}$ region was negative (red in Figure 5), $\sigma_{L}\;=\;-\sigma_{0}$, where $\sigma_{0}\;=\;0.4835$*e*/nm${}^{2}$, while the $H_{R}$ region was positive (blue in Figure 5), $\sigma_{R}\;=\;\sigma_{0}$. The limiting cases are the fully negatively (‘nn’) and positively (‘pp’) charged pores for $x_{L}\;=\;0$ and 1, respectively, while we talk about bipolar pores in between (‘np’).
 **Unipolar pores:** In the other series, one of the regions was neutral (grey in Figure 5) in the intermediate cases. These are actually two series of experiments. Starting from the ‘nn’ limiting case (from left to right in Figure 5), through unipolar ‘n0’ charge patterns, we reach the ‘00’ limiting case (neutral pore) as $x_{L}$ changes from 1 to 0. Starting from the ‘pp’ limiting case (from right to left in Figure 5), through unipolar ‘0p’ charge patterns, we reach the ‘00’ limiting case (neutral pore) as $x_{L}$ changes from 0 to 1. The ‘n0’ (‘0p’) pore, where $\sigma_{L}\;=\;-\sigma_{0}$ and $\sigma_{R}\;=\;0$ ($\sigma_{L}\;=\;0$ and $\sigma_{R}\;=\;\sigma_{0}$) exhibits rectification due to the asymmetric charge pattern.

In order to characterize charge pattern, we introduced a dimensionless net charge, *Q*, ranging from −1 to 1, defined as

(3) $$Q=x_{L}\frac{\sigma_{L}}{\sigma_{0}}+\left(1-x_{L}\right)\frac{\sigma_{R}}{\sigma_{0}}.$$

**Figure 6:**
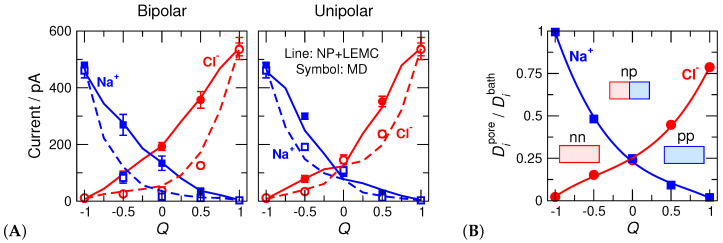
(**A**) Ionic currents as functions of *Q* for the bipolar (left panel) and unipolar (right panel) nanopores. Blue and red colors correspond to Na${}^{+}$ and Cl${}^{-}$, respectively. Symbols and lines correspond to MD and NP+LEMC results, respectively. Filled symbold and solid lines refer to the ON state (200 mV), while open symbols and dashed lines refer to the OFF state (−200 mV). (**B**) Diffusion coefficients in the pore, $D_{i}^{\mathrm{pore}}$, normalized by the bulk values, $D_{i}^{\mathrm{bath}}$, fitted to MD currents in the ON states of the bipolar pore. The fit was done for every *Q* separately.

This value is uniquely related to $x_{L}$ in the cases depicted in Figure 5. Its value is −1 for the ‘nn’ pore, 1 for the ‘pp’ pore, 0 for the ‘np’ pore, −0.5 for the ‘n0’ pore, 0.5 for the ‘0p’ pore, and 0 for the ‘np’ and ‘00’ pores. We found that the pore’s basic behavior is correlated with this parameter (Figure 6).

In order to relate our implicit-water NP+LEMC simulations to explicit-water MD simulations, we constructed an all-atom version of the model. While we did our best in building the all-atom model that is, apart from the treatment of water, is as similar to the reduced model as possible, there are differences:

- Water is explicit (SPC) in MD, while it is implicit in LEMC.
- The ions have Lennard-Jones cores in MD, while they have hard-sphere cores in LEMC.
- The pore wall is a carbon nanotube (CNT) in MD, while it is a hard wall in LEMC.
- The membrane is confined by carbon nanosheets (CNS) in MD, while with hard walls in LEMC.
- The interior of the membrane is empty (a vacuum) in MD, while it is an ϵ=78.45 region in LEMC.
- The MD simulation cell applies periodic boundary conditions, while the LEMC simulation cell is finite (a cylinder).

The most serious difference between the two systems is the treatment of water, so we consider this study as a test of the implicit-water approximation for this nanopore system.

A continuous surface charge was mimicked by placing partial point charges at the carbon atoms of the CNT. The CNT consisted of hexagons of side width 0.142 nm. There were 1682 partial charges of strength 0.0112e on the grid for the ‘pp’ pore. These same partial charges were used in the NP+LEMC calculations. This fine resolution of the pore charges was necessary, because we also compared to the PNP theory in Reference [46] (PNP results are not shown here).

The electrolyte was NaCl (for the ionic parameters see Reference [46]) at bulk concentrations 1 M. The asymmetric pores were rectifying when we applied voltages 200 and −200 mV (ON and OFF states, respectively).

#### 3.2.1. Concentration Profiles and Device Functions

**Figure 7:**
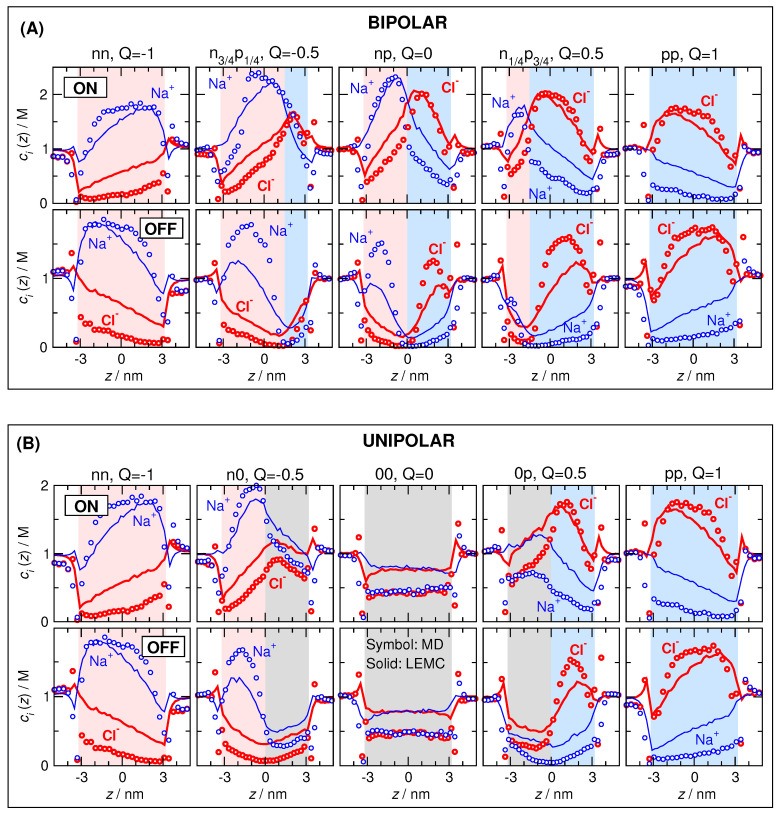
Cross-section averaged axial concentration profiles of Na${}^{+}$ (blue) and Cl${}^{-}$ (red) ions for (**A**) the bipolar and (**B**) unipolar cases. In each case, top row and bottom row show the ON and OFF states, respectively. Symbols and solid lines refer to molecular dynamics (MD) and NP+LEMC results, respectively.

The MD simulation results are our gold standard, so we fit the diffusion coefficients inside the pore, $D_{i}^{\mathrm{pore}}$, to MD current data for the bipolar pore in the ON state (Figure 6B). Because we decided to use only one adjustable parameter ($D_{i}^{\mathrm{pore}}$), it was necessary to make its value *Q*-dependent, because the pore’s behavior is severely different at different *Q* parameters as also shown by the concentration profiles (Figure 7).

As the pore charge, *Q*, increases, Na${}^{+}$ current decreases and Cl${}^{-}$ current increases (Figure 6A). One of the device functions, selectivity, changes with *Q*, with the pore being non-selective at Q=0. When the charge pattern is asymmetrical, the pore rectifies, namely, the ON current is larger than the OFF current (Figure 6A). Rectification (the other device function) has a maximum at Q≈0 in the bipolar case, while it has maxima between Q=−1 and 0 as well as between Q=0 and 1 in the unipolar case. The selectivity and rectification curves as functions of *Q* are shown in Reference [46] (their Figure 7).

The axial concentration profiles (Figure 7) determine the current, as in the case of the RyR ion channel. The major difference compared to the RyR channel is that the depletion zones have decisive roles inside the pore here, not only in the access regions as in the case of the RyR. Briefly, if an ionic species has a depletion zone somewhere inside the pore along the *z*-axis, its current is suppressed. This statement is intuitive if we imagine the pore as a collection of layers along the *z*-axis that, in turn, are imagined as resistors connected in series. If any of the resistors has a large resistance due to a depletion zone in that layer, the whole circuit has a large resistance.

We can also support our statement with a quantitative analysis. In Appendix A, we outline our slope-conductance approach that shows that the resistance of the pore is related to the integral of $c_{i}^{-1}\left(z\right)$ (Equation (A6)). Depletion zones give large contributions to that integral, and, therefore, to resistance.

#### 3.2.2. Charge Pattern Determines Device Behavior

The decisive effect of pore charge pattern does not require special verification here; the studies of Reference [46] shown in Figure 5 were devised for the purpose of studying that effect. Figure 6A for the current and Figure 7 for the concentration profiles clearly show that the charge pattern characterized by the *Q* parameter squarely determines device behavior.

When electrostatic attractions and repulsions play the primary role in forming the shape of the ionic concentration profiles—namely, defining which are the coions and which are the counterions to define where depletion zones and peaks are formed—it is not a surprise that charge pattern dominates over other factors.

#### 3.2.3. Water Molecules as Unimportant Degrees of Freedom

The decisive roles of Coulomb interactions and charge patterns also explain why water molecules can be smeared into a continuum background. Both the axial concentration profiles (Figure 7) and currents (Figure 6A) show that the device works qualitatively the same way in the case of the explicit-water (MD) and implicit-water (NP+LEMC) models.

We devoted a whole paper to this question [42], so we summarize the results of that paper. We showed that the implicit-water and explicit-water models produced qualitatively similar behavior of the current for different voltages and model parameters. Looking at the details of concentration and potential profiles, we found profound differences between the two models. However, these differences did not influence the basic behavior of the model as a device because they do not influence the *z*-dependence of the concentration profiles, which we found are the main determinants of current. Therefore, our simulations showed that reduced models can still capture the overall device physics correctly because they included the physics that is necessary from the point of view of device function. This is despite the fact that they get some important aspects of the molecular-scale physics quite wrong (e.g., radial ion packing produced by the structure of the water molecules).

#### 3.2.4. Transferability of the Fitted Diffusion Coefficient

We emphasized that it is the *qualitative* behavior that is the same on the two modeling levels. If we want *quantitative* agreement, we need to fit the parameter(s) of the reduced model to MD or to experimental data. In general, we can say that if we observe an overall *qualitative* agreement, the reduced model does its job and there is a good chance that our response function that replaces the smeared degrees of freedom is transferable. The question is what transferability means. What are the external conditions that influence the response function and what are those that do not?

This question has been already touched on with the RyR ion channel, where we stated that our choice of a single adjustable parameter (the diffusion coefficient in the selectivity filter, $D_{i}^{\mathrm{pore}}$) does not make it possible to create a response function that is transferable over charge patterns, namely, over mutations. It was, however, transferable over voltages, concentrations, and electrolyte compositions.

The situation here is the same. We attempted to create a diffusion coefficient profile that is independent of *Q*, but due to uncertainties in MD simulations and computational demand of NP+LEMC simulations, we abandoned these efforts. Instead, we realized that the difference between the MD and NP+LEMC concentration profiles (Figure 7) depends on *Q* systematically. For example, as *Q* increases, the Na${}^{+}$ profiles as obtained from MD and NP+LEMC become increasingly different. (At the same time, Cl${}^{-}$ profiles become increasingly similar.) Exactly this difference is what must be balanced by the diffusion coefficient in the pore.

Therefore, we decided to use a single $D_{i}^{\mathrm{pore}}\left(Q\right)$ value all along the pore that is allowed to vary with *Q*. We fitted $D_{i}^{\mathrm{pore}}$ to one case (bipolar/ON), and investigated transferability for the remaining three cases (bipolar/OFF, unipolar/ON, unipolar/OFF). So, we fixed the $D_{i}^{\mathrm{pore}}\left(Q\right)$ values fitted to the bipolar/ON case and used them at other cases for the same *Q*. These values are shown in Figure 6B as functions of *Q*.

As an example, let us consider *Q* values close to −1. This is close to the ‘nn’ geometry, namely, a cation selective pore. Cl${}^{-}$ ions have depletion zones in this case in both models, but they are deeper in MD than in NP+LEMC. We need, therefore, a very small $D_{{\mathrm{Cl}}^{-}}^{\mathrm{pore}}$ value to bring the NP+LEMC profiles (and, therefore, currents) down to the values yielded by MD. As *Q* increases, the difference between the Cl${}^{-}$ profiles decreases and they are pretty similar for Q=1, namely, for the ‘pp’ pore. In general, we can state that the implicit-water approximation works better (compared to MD) in the case of peaks than in the case of depletion zones.

To summarize, one job of the $D_{i}^{\mathrm{pore}}\left(Q\right)$ function is to take the differences in the explicit and implicit water models into account. The diffusion coefficient in the pore, therefore, is more than a transport coefficient that, in principle, could be calculated from autocorrelations functions or mean square displacements. It carries more information that stems from differences between the reduced model and the more realistic experimental data or MD simulations. Eventually, it is an adjustable parameter of the reduced model as a whole.

### 3.3. Selectivity Inversion Due to Charge Inversion

In the two case studies so far radial profiles were relatively unimportant. The narrow RyR pore was a crowded high density region (Figure 2) but without layering (oscillatory concentration profiles) in the radial dimension. The case of the wider nanopore in Section 3.2, however, is much more complex. The radial distribution of the ions is important because it determines the behavior of the axial profiles. This was discussed in detailed in our recent studies [50,52].

In the first study [50], we showed that bipolar nanopores exhibit a scaling behavior for a fixed σ=±1*e*/nm${}^{2}$. Specifically, we constructed a scaling parameter, $\xi \;=\;R_{\mathrm{pore}}/\lambda \sqrt{z_{+}\left|z_{-}\right|}$, where λ is the characteristic screening length of the electrolyte computer either as the Debye length (for a point-ion model) or the Mean Spherical Approximation screening length (for Primitive Model ion). (Note that screening works differently near surfaces of different curvatures (flat, concave, convex). Different equations for the capacitance can be given with an unchanged value of the Debye length. [93]) We found that for different pore sizes and different electrolyte concentrations that had the same ξ the device function (this time rectification) was the same; that is, for a given $z_{+}:z_{-}$ electrolyte, the relationship of $R_{\mathrm{pore}}$ and λ determines device behavior. If $R_{\mathrm{pore}}\ll \lambda$, the double layers formed at the nanopore’s wall in the radial dimension overlap. In that case, the counterions will be at high concentration in the middle of the pore, while coions will be at relatively low concentration. This forms depletion zones for the excluded coions. If $R_{\mathrm{pore}}\gg \lambda$, the double layers do not overlap, a bulk electrolyte is present in the pore’s center line, and depletion zones are not formed. Depletion zones are necessary for selectivity and rectification. In Section 3.2, this was not discussed because $R_{\mathrm{pore}}$ and λ (concentration) were fixed. Double layer overlap was present.

In the second study, [52] we considered the dependence of bipolar nanopores on σ for different electrolytes (1:1, 2:2, 2:1, 3:1). If multivalent ions are present, a deviation from the above scaling behavior (basically a mean-field phenomenon) appears because strong ionic correlations cause peculiar phenomena such as overcharging (overcharging means that more counterions are attracted to the surface than necessary to compensate the surface charge) and charge inversion [78] (charge inversion is the appearance of a layer of excess coions that produces a change in the sign of the electrical potential in this layer). Specifically, these correlations cause an increase in coion concentration in the second layer of ions behind the dense counterion first layer near the charged wall. Consequently, the electrostatic potential can change sign (relative to the potential at the charged wall). We showed that this accumulation of coions (anions) produces an anion leakage current, and this causes non-monotonic behavior in the device function (rectification) as σ increases. Charge inversion always manifests itself in the dimension perpendicular to the charged wall, which for pores is the radial dimension.

In this section, we present new results for the phenomenon of selectivity inversion in a negatively charged nanopore (σ=−1*e*/nm${}^{2}$) as the electrolyte is changed from 1:1 through 2:1 to 3:1. This phenomenon was shown experimentally in our paper with the group of Zuzanna Siwy [22] and interpreted with the help of GCMC simulations. It was observed that while the pore is cation selective for a 1:1 electrolyte (KCl), it becomes anion selective for a 3:1 electrolyte (CoSepCl${}_{3}$). The GCMC simulations supported the idea that the basic reason of this selectivity inversion is charge inversion. The trivalent cations stick to the negatively charged surface, overcharge it, and remain paralyzed; they do not contribute to the current significantly because their mobility near the pore is reduced by being trapped in an energy well.

Here, we show that this behavior can be reproduced without explicitly changing the mobilities of the ions (i.e., decreasing $D_{i}\left(r\right)$ near the wall) by using localized charges instead of a surface charge that is smeared over the surface relatively uniformly as it was in Section 3.2. In fact, this model is much closer to the experimental reality, because the negative charges are localized in chemical groups on the surface of an insulator, specifically, in COO${}^{-}$ groups for the PET nanopores used by Siwy et al.

Here, we show that adopting this idea can produce strong charge inversion around the binding sites now both in the *z* and *r* dimensions. The nanopore is practically the same as the one in Section 3.2: it is a cylindrical pore with $R_{\mathrm{pore}}\;=\;1$ nm and H=6 nm with c=0.1 M electrolytes on both sides (ionic radii are $R_{+}\;=\;R_{-}\;=\;0.15$ nm). We place fractional point charges on a rectangular grid on the pore’s surface of width Δz in a way that the surface charge density is kept constant at σ=−1*e*/nm${}^{2}$. Having Δz=1 nm, where −e point charges are sitting on the grid, corresponds to the experimental situation.

#### 3.3.1. Axial Concentration Profiles Determine Selectivity

**Figure 8:**
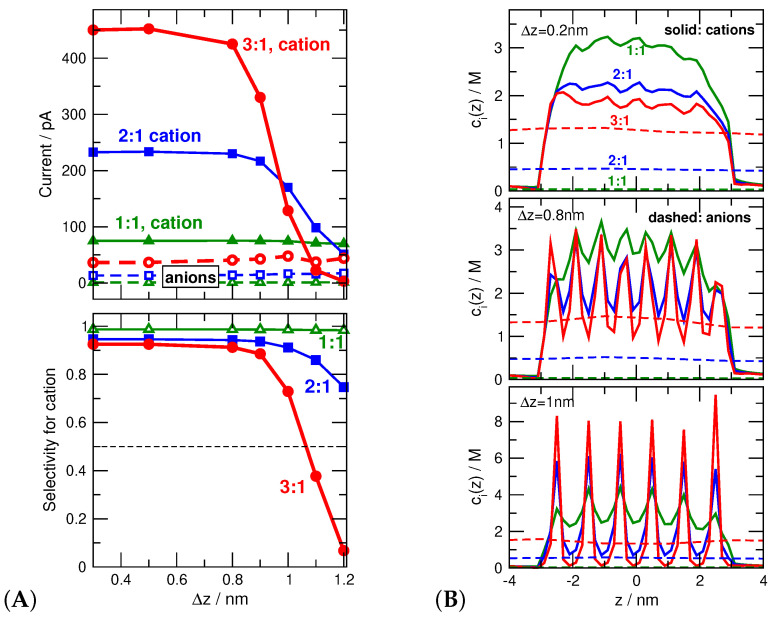
(**A**) The top panel shows ionic currents as functions of Δz for various electrolytes (green, blue, and red color refer to 1:1, 2:1, and 3:1, electrolytes). Solid and dashed lines refer to cations and anions, respectively. The bottom panel shows the cation selectivities computed as $I_{+}/\left(I_{+}+I_{-}\right)$. Values above and below 0.5 correspond to cation and anion selectivities, respectively. (**B**) Axial concentration profiles of cations (solid lines) and anions (dashed lines) in the three elecrolytes. Different panels refer to different values of Δz (0.2, 0.8, and 1 nm from top to bottom). Colors have the same meaning as in Figure 8A.

Cation selectivity defined as $I_{+}/\left(I_{+}+I_{-}\right)$ is shown in the bottom panel of Figure 8A as a function of Δz for different electrolytes (1:1, 2:1, and 3:1) for a constant $R_{\mathrm{pore}}$. The top panel shows the ionic currents from which selectivity was computed. While cation selectivity is insensitive to the fineness of the grid (the degree of localization of surface charge) in the 1:1 case, cation current (and cation selectivity with it) quickly drops as Δz increases above 0.8 nm in the 3:1 case (thick red lines).

The explanation follows from the axial cationic concentration profiles (i.e., cross-sectionally averaged concentrations) in Figure 8B. For a fine grid similar to that used in Section 3.2 and in our earlier studies (Δz=0.2 nm), [42,43,44,45,46,47,48,49,50,51,52] the cation profiles are practically constant inside the pore for all the electrolytes from 1:1 to 3:1. For the case of localized charges (Δz=1 nm), depletion zones appear along the *z*-dimension that are much deeper in the case of 2:1 and, especially, 3:1 electrolytes. As the axial depletion zones get deeper, cation currents decrease as Δz increases.

Anion currents, on the other hand, do not change significantly as Δz changes because the anion profiles do not change (Figure 8B). This statement is valid for the anion profiles too (see Figure 8B). This is not a surprise because the anions are far from the charged surface on average, so their distribution is less influenced by the localization of the pore charges. This indicates that it is the behavior of the cations that is responsible for selectivity inversion.

#### 3.3.2. Charge Localization Is an Important Degree of Freedom

The appearance of those depletion zones, however, can be fully understood only if we take into account both the *z*- and *r*-dependence of the ionic distributions. Although the statement that current primarily depends on the axial profiles remains true (Equation (A6)), understanding why the axis profiles look the way they look requires the complete picture.

**Figure 9:**
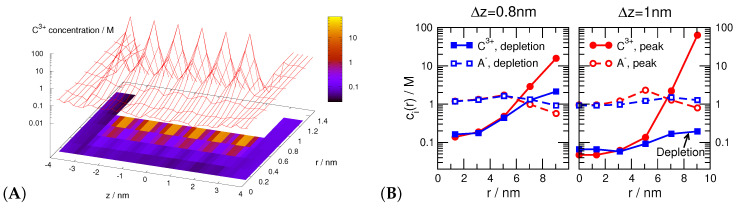
(**A**) The $c_{3+}\left(z,r\right)$ concentration profiles of the trivalent cations for the Δz=1 nm case. (**B**) Radial concentration profiles of trivalent cations (C${}^{3+}$, filled symbols with solid lines) and monovalent anions (A${}^{-}$, open symbols with dashed lines) for selected values of *z*. In the left panel (Δz=0.8 nm), the values z=0.3 nm and z=0.7 nm correspond to a peak and a depletion region, respectively. In the right panel (Δz=1 nm), the values z=0.5 nm and z=0.9 nm correspond to a peak and a depletion region, respectively.

Figure 9A shows the $c_{3+}\left(z,r\right)$ concentration profiles for trivalent cations and Δz=1 nm. The figure shows the large peaks near the localized pore charges and deep depletion zones between the peaks (note the logarithmic scale). Also, the cationic concentration profiles decline as r→0, namely, approaching the centerline of the pore.

These phenomena can be observed better if we plot the radial profiles for fixed *z* values that correspond to either a peak (red) or a depletion region (blue). (For the actual values of *z*, see the caption of Figure 9B.) The left panel of Figure 9B shows radial profiles for Δz=0.8 nm; the corresponding axial profiles were shown in the middle panel of Figure 8B. The important thing to note is that the radial profiles do not differ much for different values of *z*. Depletion zones, therefore, are not formed in this case (see solid blue line with filled squared). It is important to point out that charge inversion is present in this case in the radial profiles; the anion profiles are larger in and around the center line of the pore. It is, however, only present in the radial direction.

The right panel of Figure 9B shows the radial profiles for Δz=1 nm. This small difference in Δz results in a significant change in the behavior of the ions. The cation profiles show the large peaks for z=0.5 nm (solid red line with filled circles), while they exhibit depletion zone for z=0.9 nm (solid blue line with filled squares). This different behavior at z=0.5 nm and z=0.9 nm produces the oscillating axial concentration profiles with the axial depletion zones of Figure 8B. In this case, therefore, we have charge inversion in both the radial and axial directions.

Taken together, these results show that the way we place the pore charges on the wall matters from the point of view of reproducing device function (pore selectivity and specifically its change due to charge inversion). Specifically, modelers probably need to step beyond the continuous surface charge distribution and to build localized pore charges into the reduced model. Counterion interactions with pore charges depend on the distance from a local binding site in all directions. In the case here, charge inversion around a local binding site produced important axial depletion zones. However, even when not considering cases with charge inversion, different ion correlations around localized pore charges can potentially produce similar important axial effects that are missed with a uniformly charged wall.

#### 3.3.3. Future Work

While it is clear that the location and discreteness of pore charges are an important degree of freedom, whether we need to use explicit particles to model the atoms of the COO${}^{-}$ groups is a subject of ongoing research. We suspect it is not vital since the charge inversion at the core of the device behavior is a product of charge itself, not the shape or mobility of the atoms producing the charge.

Also, we do not know whether we need to change the diffusion coefficient, $D_{i}\left(z,r\right)$, in the radial dimension in order to fit to experiments or to dynamic simulations. Work is currently underway with all-atom MD simulations to determine this.

## 4. Conclusions

In reduced models, some degrees of freedom (the important ones) are modeled explicitly, while the rest (the unimportant ones) are taken into account implicitly in some way, via response functions, for example. Before the age of computers, all models were reduced. When MD simulations became an everyday computational tool, atomic models became the new standard in certain areas of chemistry, physics, and biology. While understanding nanoscale physics is vital, we believe that the ease of use of MD has sometimes caused the baby to be thrown out with bath water. Rather, we think that what is needed are clever models that are necessarily reduced to some degree to be computationally feasible.

Modeling of ion channels and synthetic nanopores is a case in point. This modeling is inherently difficult as nanoscale interactions and physics directly translate into measurable phenomena (what we call device functions). By simplifying the physics to be modeled, reduced models have a number of advantages over all-atom simulations. However, building such models is in many ways more art than science. Here, we have taken both old and new data from our simulations of ion channels and nanopores and distilled from them four rules of thumb (principles) for constructing reduced models for nanopores. These are

1. The current carried by an ionic species is mainly determined by the axial concentration profile of that species inside the pore.
2. Care must be taken to model the pore charges since they produce the local ion concentrations, and, consequently, device function.
3. The important degrees of freedom that must be included in the model are those that depend on the input parameters of the device (voltage and concentration), while those that do not can be replaced by response functions.
4. Having the parameters within a response function not depend on external conditions (or at least have minimal dependence) makes those parameters transferable to other conditions, and this makes it possible for the model to make predictions that can be tested.

Our goal is to offer insights into how to think about reduced model, but also to point out the subtleties and consequences of the choices a modeler might make. Specifically, for each rule of thumb we showed that its interpretation is not as straightforward as it might seem. For example, while large ion concentrations are important, so are areas with small concentrations which act as large resistors that can dominate the current. Also, charged groups seemingly far from the key locations (e.g., the selectivity filter of an ion channel) can grossly change current/voltage curves. Overall, testing and probing to find the important degrees of freedom that capture the axial-direction physics is the key to reproducing device function and understanding the physics behind the device function; for example, using uniform versus discrete pore charges can have measurable consequences. Once these have been identified, approximating other physics as response functions is a lot easier.

Lastly, we note that while reduced models are important to understand these devices, they are only one part of the continuum of modeling levels that are possible. All-atom and even quantum mechanical simulations play key roles as well in defining the physics of nanopores at the atomic and molecular levels. The role of reduced models is on a larger scale, namely to identify the physics of the device as a whole using the nanoscale physics defined at more detailed levels of modeling. They are the last step to couple atoms to experimental measurements. 

## Figures and Tables

**Table 1:** Parameters of ions as used in the NP+Local Equilibrium Monte Carlo (LEMC) simulations. The last column shows the density functional theory (DFT) value $D_{i}^{\mathrm{pore}}$, the diffusion coefficient in the selectivity filter, for comparison; the values for the vestibules are found in Reference [16]. ${}^{a}$ This value was not fitted due to the fact that the channel does not let Cl${}^{-}$ through.

Ion	$R_{i}$ (Pauling)	$D_{i}^{\mathrm{bulk}}$	$D_{i}^{\mathrm{pore}}$ (LEMC)	$D_{i}^{\mathrm{pore}}$ (DFT)
	nm	$10^{-9}$ m${}^{2}$s${}^{-1}$		
Na${}^{+}$	0.095	1.334	0.141	0.0365
Cs${}^{+}$	0.169	2.056	0.193	0.0418
Ca${}^{2+}$	0.99	0.792	0.0243	0.0041
Cl${}^{-}$	1.81	2.032	$0.25{\;}^{a}$	0.02

## Supplementary Materials

The following are available online at https://www.mdpi.com/1099-4300/22/11/1259/s1.

## Appendix A. Slope Conductance Theory

Let us assume rotational symmetry, so quantities depend on the variables z,r. Let assume, furthermore, that μ does not depend on *r*; results (not shown) support this assumption. Let us integrate the NP equation (Equation (1)) over the cross section:

(A1) $$I_{i}=-z_{i}e\int_{A(z)}j_{i}\left(z,r\right)da=\frac{z_{i}e}{kT}\left[\int_{A(z)}D_{i}\left(z,r\right)c_{i}\left(z,r\right)da\right]\frac{d\mu_{i}\left(z\right)}{dz}=\frac{z_{i}e}{kT}N_{i}\left(z\right)\frac{d\mu_{i}\left(z\right)}{dz}$$

for any *z* inside the pore with

$$N_{i}\left(z\right)=\int_{A(z)}D_{i}\left(z,r\right)c_{i}\left(z,r\right)da.$$

Let us rearrange and integrate over the pore

(A2) $$I_{i}\int_{H_{1}}^{H_{2}}\frac{dz}{N_{i}\left(z\right)}=\frac{z_{i}e}{kT}\int_{H_{1}}^{H_{2}}d\mu_{i}\left(z\right)=\frac{z_{i}e}{kT}\Delta \mu_{i}.$$

If we assume that bulk concentrations are the same on the two sides of the membrane, the electrochemical difference is

(A3) $$\Delta \mu_{i}=z_{i}eU,$$

where *U* is the voltage across the pore. Substituting into Equation (A2), we obtain that

(A4) $$I_{i}\int_{H_{1}}^{H_{2}}\frac{dz}{N_{i}\left(z\right)}=\frac{z_{i}^{2}e^{2}}{kT}U$$

from which the resistance (the reciprocal of conductance) is obtained as

(A5) $$\frac{1}{G_{i}}=\frac{U}{I_{i}}=\frac{kT}{z_{i}^{2}e^{2}}\int_{H_{1}}^{H_{2}}\frac{dz}{N_{i}\left(z\right)}.$$

If we assume that $D_{i}^{\mathrm{pore}}\left(z\right)$ does not depend on *r* inside the pore, we can write that

(A6) $$\frac{1}{G_{i}}=\frac{kT}{z_{i}^{2}e^{2}}\int_{H_{1}}^{H_{2}}\frac{dz}{D_{i}^{\mathrm{pore}}\left(z\right)A\left(z\right)c_{i}\left(z\right)},$$

where $c_{i}\left(z\right)=\frac{1}{A(z)}\int_{A(z)}c_{i}\left(z,r\right)da$ is the radially-averaged concentration. If $c_{i}\left(z\right)$ is very small somewhere in the pore along the *z*-axis, the integral, the resistance, becomes large. This analysis was used in several works [17,18,21,23,24,27,46,48,52] to relate concentration profiles to currents.
