# Supplementary material for: Modeling the Device Behavior of Biological and Synthetic Nanopores with Reduced Models
Source: Entropy (Basel). 2020 Nov 5;22(11):1259. doi: 10.3390/e22111259 (PMC7711659; doi:10.3390/e22111259)
Supplement: Supplementary file 1 [file entropy-22-01259-s001.pdf]

Supplementary Material

Modeling the device behavior of biological and synthetic nanopores with reduced models

Dezső Boda[1,†]*, Mónika Valiskó[1,†], and Dirk Gillespie[2,†]

[1] Department of Physical Chemistry, University of Pannonia, P.O. Box 158, H-8201 Veszprém, Hungary
[2] Department of Physiology and Biophysics, Rush University Medical Center, Chicago, Illinois 60612, USA
* Correspondence: boda@almos.uni-pannon.hu; Tel.: +36-88-624-000/6041 (D.B.)
† These authors contributed equally to this work.

Version October 12, 2020 submitted to Entropy

Abstract: The goal of this Supplementary Material is to support the idea of modeling the ryanodine receptor (RyR) calcium channel with a reduced model beyond the results presented in the main text.

Keywords: nanopores, ion channels, reduced models, Monte Carlo, classical Density Functional Theory, Poisson-Nernst-Planck

---

In the main text, we showed results for simulations of RyR using the NP+LEMC method. In this Supplementary Material, we show more results and comparisons to experimental data. The simulation results shown here were performed for a smaller system size. The fitted diffusion coefficients, therefore, are different here than in the main text, where the simulations were redone with a larger system size, and the diffusion coefficients fit accordingly. This does not change the qualitative conclusions; the reduced model is robust in both cases according to the rules of thumb described in the paper. The diffusion coefficients of these simulations are shown in Table 1. The model is shown in Fig. 1 of the main text.

Table 1. Parameters of ions as used in the NP+LEMC simulations. Units are nm for R_i and 10^{-9} m^2s^{-1} for the diffusion coefficients. The last column shows the DFT value D_i^{pore}, the diffusion coefficient in the selectivity filter, for comparison; the values for the vestibules are found in Ref. [1].
[a] This value was not fitted due to the fact that the channel does not let Cl^- through.

Ion	R_i (Pauling)	D_i^{bulk}	D_i^{pore} (LEMC)	D_i^{pore} (DFT)
Li^+	0.665	1.029	0.0446	0.0129
Na^+	0.095	1.334	0.127	0.0365
K^+	1.33	1.849	0.257	0.0365
Rb^+	1.49	2.072	0.233	0.0592
Cs^+	0.169	2.056	0.174	0.0418
Ca^{2+}	0.99	0.792	0.0127	0.0041
Mg^{2+}	0.65	0.706	0.0135	0.0042
Cl^-	1.81	2.032	0.25[a]	0.02

The results shown here are a sample of all the simulations that were performed. We have, in fact, simulated all the experiments that were already reproduced by the 1D DFT model. [1–5] Here, we focus on the results of Refs. [1] and [6]. The original sources of the data can be found in Ref. [6]

Our general conclusion is that the 3D model works as well as the 1D model except for the mutants (see Fig. 6), as discussed in the main text. The sample here shows the power of the reduced model. The figures are discussed in their captions.

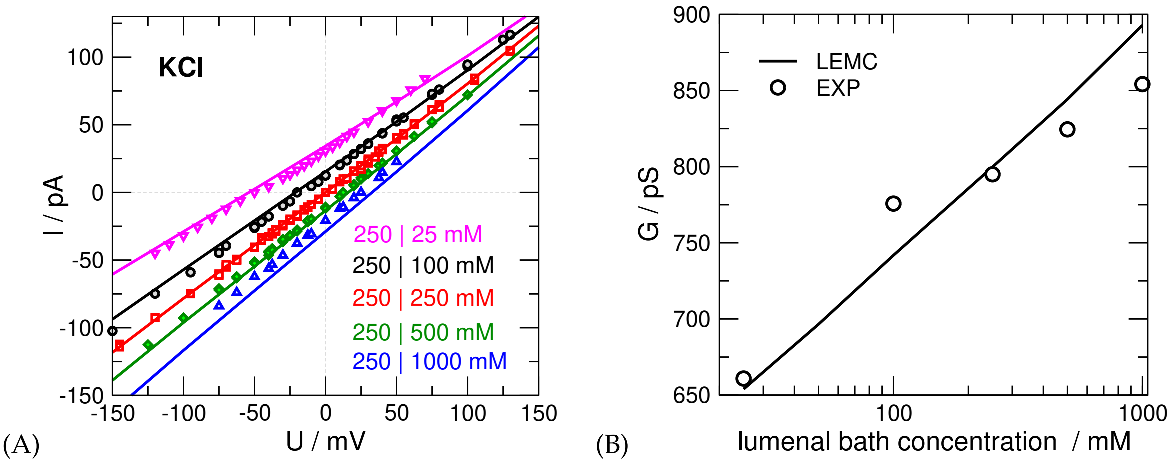

Figure 1. (A) IV curves for KCl with different KCl concentrations on the luminal side while the cytosolic concentration is fixed at 250 mM. Here and in the remaining figures, symbols and lines represent experimental and NP+LEMC data, respectively, unless otherwise specified. The curves are shifted due to changing asymmetry in bath concentrations on the two sides of the membrane (Nernst equation) with a corresponding change in the reversal potential. (B) The conductance of the RyR channel as a function of the luminal KCl concentration. The slopes of the curves in panel (A) also change with the luminal KCl concentration. The slope (computed from a liner fit) is plotted in panel (B). This plot also shows the transferability of $D_{K^+}^{pore}$ over bath concentrations. This value was fit to the 250 | 250 mM case and was used in all other cases.

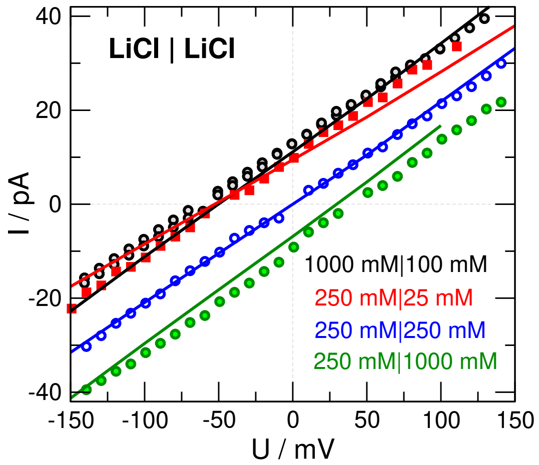

Figure 2. IV curves for LiCl with different concentrations on the luminal side. The conclusions are similar to those drawn at Fig. 1 for KCl. The 1000 | 100 mM and 250 | 25 mM cases refer to the same luminal | cytosol concentration ratio. Accordingly the IV curves for these two cases are quite close to each other.

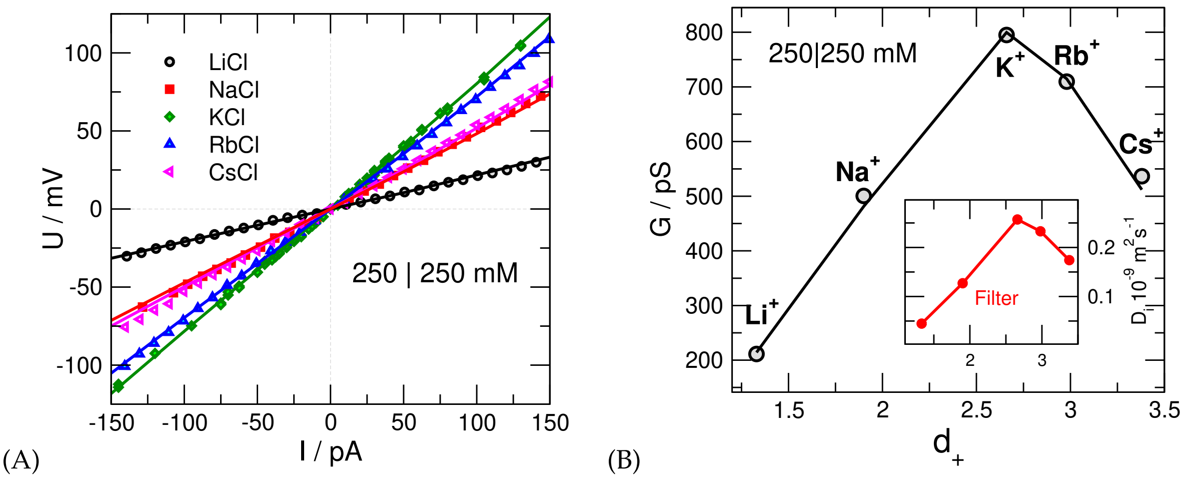

(A) (B)

Figure 3. (A) IV curves for 1:1 electrolytes with different cations for a symmetric (250 | 250 mM) case. The agreement between experiment and simulations are good because the diffusion coefficients were fitted to this case for U=100 mV. It should be noted that the seemingly simple linear IV curves (especially at large applied potentials) are actually a nontrivial output of the simulations. Gillespie [6] showed that the IV curves are nonlinear unless all the pore charges are included. (B) The conductance as a function of the cation diameter. The conductance follows the behavior of D_i^{pore} (see inset).

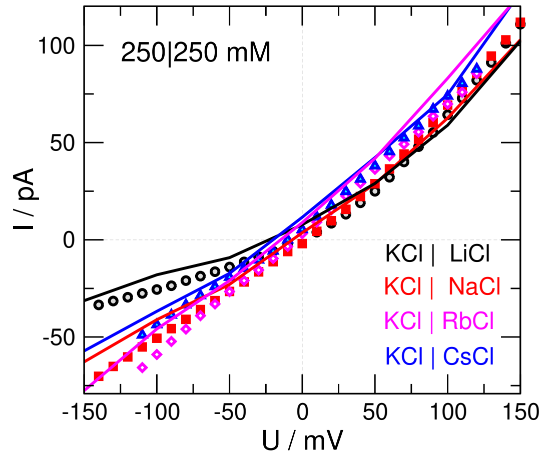

Figure 4. IV curves for biionic conditions with 250 mM KCl on the cytosolic side and with various salts, as at 250 mM, on the luminal side.

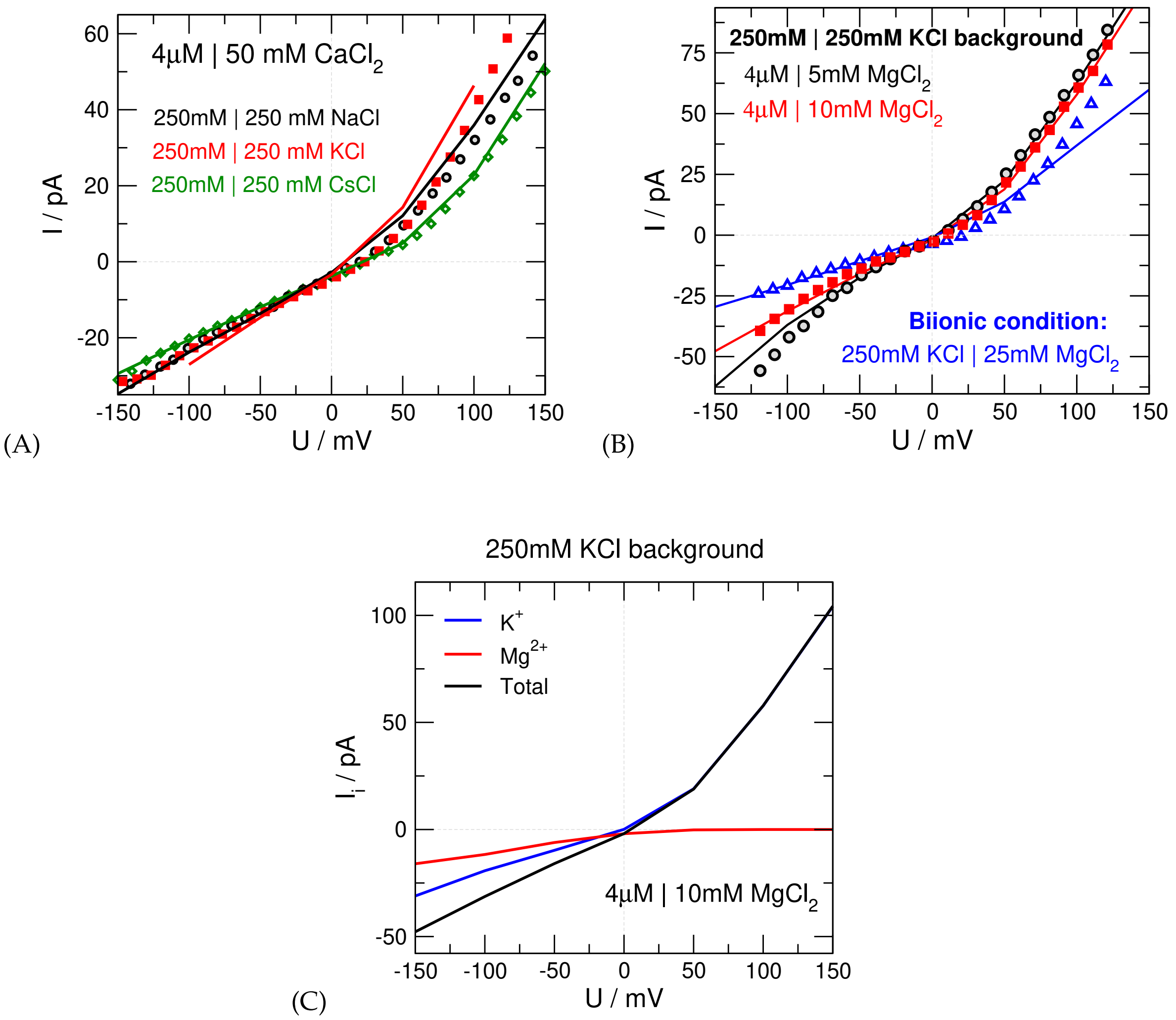

Figure 5. (A) IV curves for $CaCl_2$–monovalent salt mixtures. $CaCl_2$ is mainly on the luminal side, while different 1:1 salts (NaCl, KCl, CsCl) are symmetric (250 | 250 mM). These results show the transferability of the $D^{\mathrm{pore}}_{\mathrm{Ca}^{2+}}$ coefficient (which was fitted to a $CaCl_2$–KCl mixture) to other 1:1 backgrounds. (B) Various KCl–$MgCl_2$ mixtures with two cases. The first case (black and red) is when KCl is a background and $MgCl_2$ is added to the luminal side in increasing amount (5 and 10 mM). The second (blue) is biionic case when KCl is on the cytosolic side and $MgCl_2$ is on the luminal side. (C) Divalent vs. monovalent competition can be better characterized shown the currents carried by individual ionic species. The IV curve for the 10 mM $MgCl_2$ in 250 mM KCl background case (red in panel (B)) is shown in black. Species ion currents are shown in blue (K^+) and red (Mg^{2+}). At positive voltages the current is purely K^+ current because Mg^{2+} does not flow from the cytosolic side. The case of negative voltages is the competitive situation. Now, a portion of the total current is carried by Mg^{2+}. (D) The biionic case (blue in panel (B)) is shown in black, with species ion currents shown in the color scheme of panel (C). There is no such a competition at large voltages. At small voltages, however, competition occurs; K^+ and Mg^{2+} ions compete for space in the channel and their currents are predominantly driven by their concentration gradients.

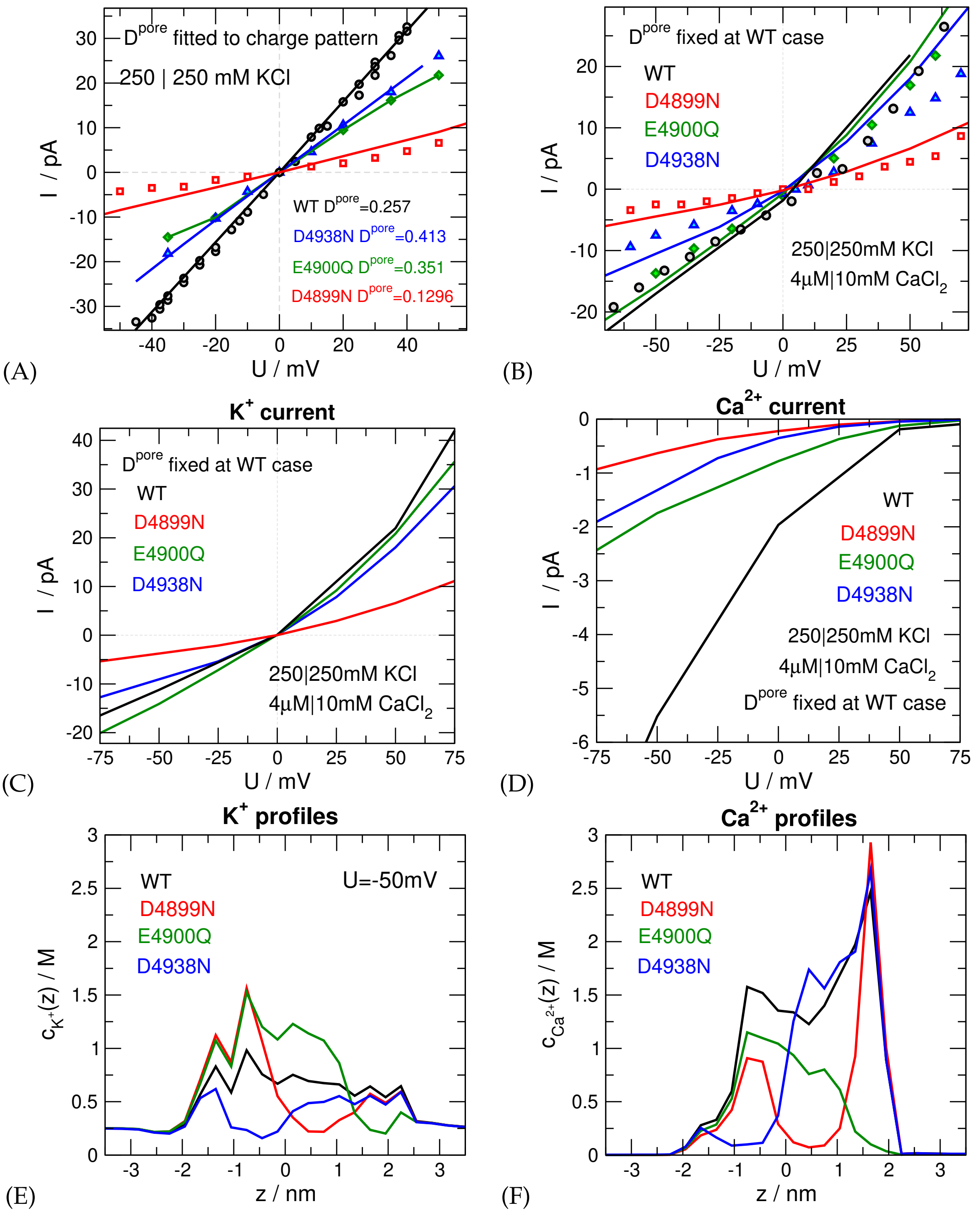

Figure 6. (A) IV curves for mutants in symmetric 250 mM KCl. The $D_{K^+}^{\mathrm{pore}}$ diffusion coefficients have been refitted (see legend). Deviations in the fitted values show that $D_{K^+}^{\mathrm{pore}}$ is not a transferable parameter over various charge patterns (see main text). The largest reduction of current occurs for the D4899N mutant, when the D4899 amino acids are changed to uncharged Asparagine (N). The Q and N amino acids are modeled as uncharged oxygens. (B) IV curves for mutants with 10 mM $CaCl_2$ on the luminal side and with symmetric 250 mM KCl as a background. The diffusion coefficients have *not* been refitted for the mutants in this case. (C) K^+ currents and (D) Ca^{2+} currents comprising the total currents of panel (B). As charged groups are taken away from the pore, the channel becomes less Ca^{2+} selective (at negative voltages). K^+ currents are generally larger than Ca^{2+} currents because of the larger bath K^+ concentrations (250 mM vs. 10 mM). The affinity of the channel, however, is much larger for Ca^{2+}, as shown by panels (E) and (F) through concentration profiles.

1. Gillespie, D. Energetics of Divalent Selectivity in a Calcium Channel: The Ryanodine Receptor Case Study. *Biophys. J.* **2008**, *94*, 1169–1184. doi:10.1529/biophysj.107.116798.
2. Gillespie, D.; Fill, M. Intracellular Calcium Release Channels Mediate Their Own Countercurrent: The Ryanodine Receptor Case Study. *Biophys. J.* **2008**, *95*, 3706–3714. doi:10.1529/biophysj.108.131987.
3. Gillespie, D.; Giri, J.; Fill, M. Reinterpreting the anomalous mole fraction effect: The Ryanodine receptor case study. *Biophys. J.* **2009**, *97*, 2212–2221. doi:10.1016/j.bpj.2009.08.009.
4. Gillespie, D.; Chen, H.; Fill, M. Is ryanodine receptor a calcium or magnesium channel? Roles of K^+ and Mg^{2+} during Ca^{2+} release. *Cell Calcium* **2012**, *51*, 427–433. doi:10.1016/j.ceca.2012.02.001.
5. Gillespie, D.; Xu, L.; Meissner, G. Selecting Ions by Size in a Calcium Channel: The Ryanodine Receptor Case Study. *Biophys. J.* **2014**, *107*, 2263–2273. doi:10.1016/j.bpj.2014.09.031.
6. Gillespie, D.; Xu, L.; Wang, Y.; Meissner, G. (De)constructing the Ryanodine Receptor: Modeling Ion Permeation and Selectivity of the Calcium Release Channel. *J. Phys. Chem. B* **2005**, *109*, 15598–15610. doi:10.1021/jp052471j.

© 2020 by the authors. Submitted to *Entropy* for possible open access publication under the terms and conditions of the Creative Commons Attribution (CC BY) license (http://creativecommons.org/licenses/by/4.0/).
